# Editorial: The Physiological and Genetic Influences of Meditation and Tai Chi on Mental, Emotional, and Movement Regulation

**DOI:** 10.3389/fphys.2020.581841

**Published:** 2020-10-23

**Authors:** Yi-Yuan Tang, Marjorie Woollacott

**Affiliations:** ^1^Department of Psychological Sciences, Texas Tech University, Lubbock, TX, United States; ^2^Department of Human Physiology, University of Oregon, Eugene, OR, United States

**Keywords:** meditation, Tai Chi, physiology, genetic influences, brain, self-regulation, bodifulness, mindfulness

Growing evidence shows the efficacy of mind-body interventions such as meditation and Tai Chi and suggested underlying mechanisms (Gatts and Woollacott, 2007; Tang et al., 2015; Wu et al., 2018; Liu et al., 2019; Cheng et al., 2020). However, little is known about their physiological and genetic influences on mental, emotional, and movement regulation.

Tai Chi is a slow, gentle mind-body practice facilitating sensory awareness of the speed, force, and execution of movement. In practice, the body is naturally relaxed and extended, the mind is calm but alert, and body postures and movements are coordinated. Tai Chi practice involves balanced postures that flow from one to another and promote concentration and mindfulness to improve motor efficiency, flexibility, and optimize motor control (Li et al., 2001). These components can be termed bodifulness and mindfulness (Tang et al., 2015, 2019).

In this Research Topic, we collected recent findings using unique and well-validated measures in healthy and patient populations. Using a classical reach-to-grasp task, Sartori et al. showed that a Tai Chi group improved motor efficiency and flexibility compared to untrained controls. Mild cognitive impairment (MCI) reduces finger tapping performance (FTP), an index of motor function. Zhang et al. used infrared photoelectric sensing to detect FTP in elderly groups—healthy vs. MCI subjects with/out Tai Chi practice. Results showed that FTP in both hands in MCI subjects was lower than in healthy subjects. However, in the Tai Chi group, only the dominant right hand's FTP of MCI subjects was lower than in healthy subjects; left hand's FTP remained normal, suggesting Tai Chi could increase motor function in MCI subjects. Major depressive disorder (MDD) accompanies depressed mood and low vitality/fatigue. Xu et al. showed that Tai Chi improved mood and vitality in MDD, accompanying insular functional connectivity changes via fMRI related to mood/fatigue improvements. Tai Chi also has protective effects on coronary heart disease (CHD). At the molecular level, serum miR-24 and miR-155 levels relate to CHD severity. Li et al. reported that Tai Chi improved CHD prognosis, affecting serum levels of miR-24 and miR-155.

Mindfulness meditation (MM) is popular but its definition is in debate. Some claim that mindfulness-based stress reduction (MBSR) is an exemplar of MM that fosters mindfulness, but other variations are interventions that incorporate mindfulness as just one component (Creswell, 2017). However, this might be misleading since MBSR consists of multiple components (Kabat-Zinn, 1990; Davidson and Kabat-Zinn, 2004; Smith, 2004; Tang et al., 2017). For example, Valim et al. compared one session of a positive emotion-based meditation focused on gratitude (EBM) and classical MM using an emotional regulation task and found that EBM outperformed MM. Notably, EBM included the practice of mindfulness and added emotional-regulation training. Therefore, the term mindfulness meditation should not define the nature of a program. Instead, the exact components and instructions of mindfulness practice are the key. For details on meditation-related outcomes, please see reviews (Chiesa and Malinowski, 2011; Hölzel et al., 2011; Goyal et al., 2014; Tang et al., 2015; Black and Slavich, 2016; Russell-Williams et al., 2018).

In summary, this Research Topic covers the main forms of mind-body practice to reveal effects at psychological, physiological, neurobiological, and molecular levels, resulting in deepening our understanding of underlying mechanisms and providing new insights for developing appropriate behavioral interventions.

## Author Contributions

Y-YT and MW drafted, revised, and finalized the manuscript.

## Conflict of Interest

The authors declare that the research was conducted in the absence of any commercial or financial relationships that could be construed as a potential conflict of interest.
